# Drug resistance genomics of the antimalarial drug artemisinin

**DOI:** 10.1186/s13059-014-0544-6

**Published:** 2014-11-25

**Authors:** Elizabeth A Winzeler, Micah J Manary

**Affiliations:** School of Medicine, University of California San Diego, Gilman Drive, La Jolla, CA 92093 USA

## Abstract

Across the globe, over 200 million annual malaria infections result in up to 660,000 deaths, 77% of which occur in children under the age of five years. Although prevention is important, malaria deaths are typically prevented by using antimalarial drugs that eliminate symptoms and clear parasites from the blood. Artemisinins are one of the few remaining compound classes that can be used to cure multidrug-resistant *Plasmodium falciparum* infections. Unfortunately, clinical trials from Southeast Asia are showing that artemisinin-based treatments are beginning to lose their effectiveness, adding renewed urgency to the search for the genetic determinants of parasite resistance to this important drug class. We review the genetic and genomic approaches that have led to an improved understanding of artemisinin resistance, including the identification of resistance-conferring mutations in the *P. falciparum kelch13* gene.

## Introduction

**Figure 1:**
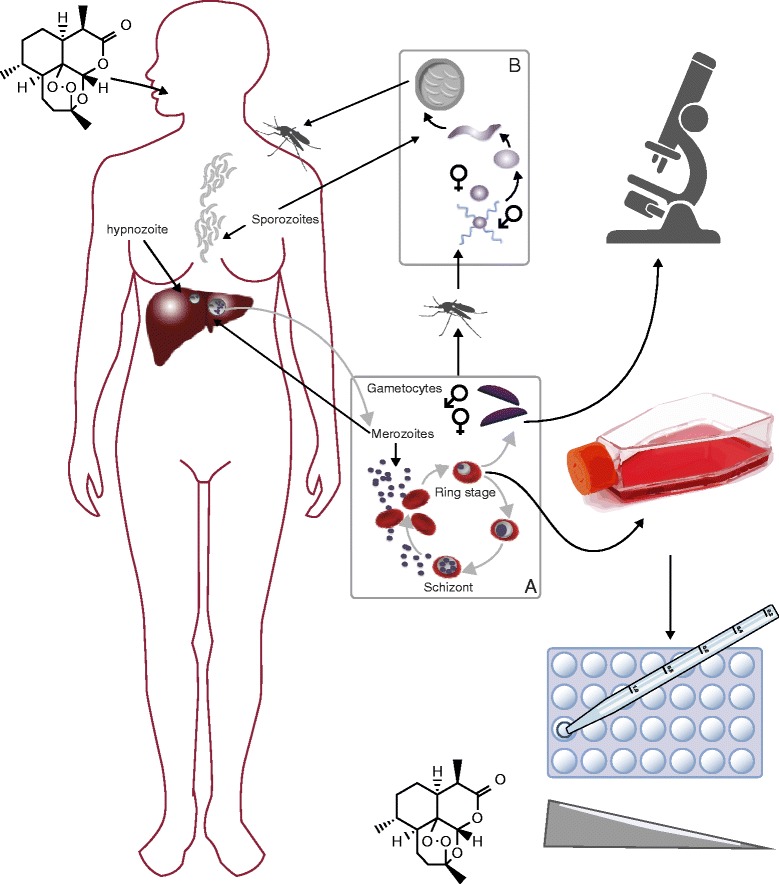
**The lifecycle of** ***Plasmodium*** **that begins with the bite of a female** ***Anopheles*** **mosquito which releases infective sporozoites into the blood of the host.** The sporozoites travel to the liver and invade liver cells. Within the liver the sporozoites mature into schizonts, which subsequently grow and produce haploid forms called merozoites. In *P. vivax*, these liver forms can remain dormant for years as hypnozoites and cause relapse of infection. Merozoites re-enter the bloodstream and invade red blood cells and undergo a cycle of asexual multiplication (A); however, some of the merozoites develop into sexual forms called gametocytes, which circulate in the bloodstream, and can be ingested by a mosquito, thus continuing the malaria lifecycle (B). Drug efficacy can be monitored by collecting blood samples in a treated patient and counting the number of infected erythrocytes using microscopy, or the parasites can be taken into long-term or short-term tissue culture, and these cultured parasites can be mixed with a drug at different concentrations and their *in vitro* survival or growth monitored [51]. The grey arrows depict the progression of the *Plasmodium* lifecycle and the black arrows indicate lifecycle forms.

Malaria, classically identified by paroxysm, fever, and flu-like symptoms recurring in 48- or 72-hour cycles, is caused by protozoan parasites of the genus *Plasmodium* and is transmitted by the bite of female *Anopheles* mosquitoes (Figure 1). The species causing the most severe form of the disease is *Plasmodium falciparum* (Box 1). Although insecticide-treated bed nets, and other preventive measures are important in the control of malaria, in the absence of a licensed vaccine and acquired, fully-protective immunity, chemotherapy has been and continues to be one of the best ways to prevent deaths, to control symptoms, and to eliminate parasites from a given geographic region. A recurrent problem with chemotherapy is that parasites, like other microbes, can and will rapidly evolve mechanisms to escape drug pressure and survive. Although arguably augmented by other factors, such as reduced spending on malaria control, the emergence and spread of multidrug-resistant *P. falciparum* parasites has probably contributed, directly or indirectly, to hundreds of millions of new cases each year and to millions of unnecessary deaths between 1970 and 2000 [1-3].

**Figure 2:**
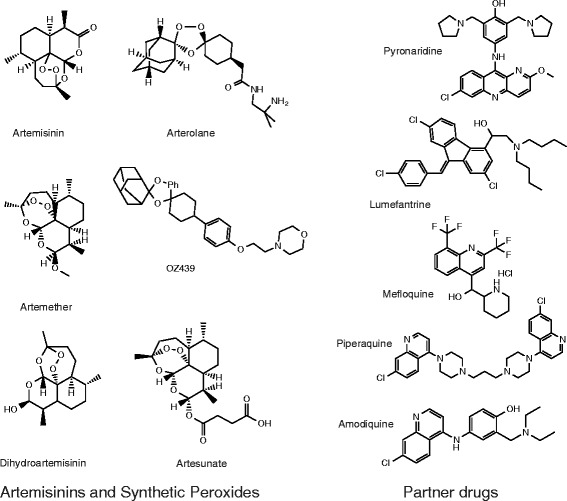
**Chemical structures of commonly used antimalarials, including artemisinin, artesunate, artemether and OZ439.** The chemistry of artemisinins is described in detail in Box 2.

The introduction of a new antimalarial treatment has been soon followed by the emergence of resistance to that treatment; perhaps most notably, parasites resistant to the antimalarial atovaquone were discovered the same year the drug was introduced [4]. After *P. falciparum* parasites became resistant to chloroquine, pyrimethamine/sulfadoxine, mefloquine and then atovaquone, *P. falciparum* malaria became very difficult to cure. Thus, the world enthusiastically welcomed the appearance of a new class of medicines based on extracts from the sweet wormwood plant, *Artemisia annua*. The antimalarial activity of *A. annua* had been rediscovered in a screen of traditional medicines for those able to cure mice and monkeys that had rodent and simian malaria, respectively (reviewed in [5,6]). Artemisinin derivatives (Figure 2) are typically combined with a partner drug, typically from a chemical family such as the aryl alcohols or 4-aminoquinolones, to comprise artemisinin-based combination therapies (ACTs). Indeed, the World Health Organization only supports the use of artemisinins in combinations, reasoning that this will delay the appearance of drug resistance because a parasite will need to acquire resistance to two drugs as opposed to just one. Although not recommended for use everywhere, ACTs are currently considered the most effective treatments for *P. falciparum* malaria in areas where drug resistance to other therapies has been a problem. Nevertheless, clinical trials from Southeast Asia indicate parasites have now acquired resistance to artemisinin-based monotherapies and some ACTs appear to be losing effectiveness [7]. Although no deaths can be directly attributed to resistance, further reductions in ACT efficacy could result in malaria again becoming a possibly incurable and often fatal disease.

Later-stage clinical trials on novel classes of antimalarial compounds [8-10] to replace artemisinin are currently underway, but no new drugs are expected to be licensed within the next few years. In the absence of a forthcoming replacement medicine, concerned physicians, scientists, and government officials have been working diligently to try to find parasite genetic markers that predict artemisinin resistance. Such markers will facilitate tracking of the spread of resistance and hopefully will allow resistance to be contained before early-stage treatment failures and possible deaths result. Importantly, having a genetic marker could also prevent deaths: if surveillance identifies the widespread presence of resistance-associated alleles in a given geographic region, patients in those regions might be admitted to a hospital for closer observation during treatment or given alternative therapies. In this review, we describe the genomic detective work that has been used to find the genes involved in artemisinin resistance, the emerging evidence that mutations in a gene encoding a Kelch-propeller domain protein confers resistance in *P. falciparum* malaria, alternative hypotheses, and the open questions that remain.

## Hypotheses about artemisinin’s function and its possible association with resistance

A logical place to look for genes that are involved in resistance to a particular drug would be in the molecular pathways associated with the target. For example, resistance to antifolate drugs is often caused by mutations in proteins in the folate biosynthesis pathway. Surprisingly, even though 331 million courses of various ACT treatments were given in 2013 [2], the mechanisms by which artemisinins act is still the subject of intensive investigation (Box 2; reviewed in [11]). In 2003, it was reported that artemisinin targets the *P. falciparum* homolog of the sarcoplasmic reticulum calcium-transporting ATPase (SERCA), PfATP6 [12]. This hypothesis was based on evidence that artemisinin decreased ATPase activity in *Xenopus* oocytes expressing PfATP6 with similar potency to thapsigargin, another SERCA inhibitor. The hypothesis was subject to substantial investigation, but no further association between *pfatp6* mutations and artemisinin could be firmly established [13-16]. Importantly, several years later, it was eventually shown that, when compared with isogenic controls, no novel *pfatp6* mutations were present in Asian *P. falciparum* parasites that had acquired resistance to artemisinin [17], nor were there mutations in rodent malaria parasites that were resistant to artemisinin and artesunate [16].

## Known multidrug-resistance genes

Some of the original studies seeking to find genes that are involved in artemisinin resistance were based on the hypothesis that known transporter-encoding genes, including *pfmdr1* [18,19], encoding the *P. falciparum* Multidrug resistant protein 1 (PfMDR1), and *pfcrt* [20], encoding the *P. falciparum* Chloroquine resistance transporter (PfCRT), would contribute to resistance. Some evidence has been encouraging: decreasing the number of copies of the gene encoding PfMDR1 resulted in increased sensitivity to artemisinin [21]. Mutations in *pfmdr1* have also been shown to modulate inhibition constant 50 (IC_50_) values for artemisinin *in vitro* [22]. Furthermore, field studies revealed an overrepresentation of the *pfmdr1* I876V mutation in parasites causing recurrent infections after artemether-lumefantrine treatment in Africa [23]. Prevalent mutant forms of *pfcrt* that confer chloroquine resistance have been shown to increase parasite susceptibility to artemisinin, and epidemiological studies have shown selection for wild-type *pfcrt* in endemic settings where ACTs are used [24,25]. Despite frequent reports of possible associations, the genotyping of parasites in Cambodia, where artemisinin resistance now appears most widespread and where monotherapies were used, have indicated that specific coding changes in these candidate genes as well as in *pfatp6* and *pfubp1* are not strongly correlated with resistance [26]. On the other hand, many of these assays looked for single nucleotide variants (SNVs) in the gene and may not have detected an increase in copy number. The lack of disease association with specific genes in Asia does not necessarily mean that these genes do not contribute to resistance or give a different resistance phenotype.

## Evolution studies

In other microbial systems, genes that are involved in resistance have been identified by first creating resistant mutants and then mapping the genes that confer resistance. Such an approach would be attractive except that crosses in *P. falciparum* have required the use of chimpanzees, in which progeny parasites are grown and cloned after they emerge from the liver after a genetic cross. *Plasmodium chabaudi* rodent parasites can be genetically crossed more readily in the laboratory (Box 1), and hence researchers were able to create *P. chabaudi* rodent parasites that were resistant to artemisinin and artesunate by continuing exposure to sublethal concentrations of these compounds [16,27]. They then crossed the ART resistant line to another sensitive line of parasites that differed from the original at many genetic positions. Instead of cloning out and genotyping the recombinant progeny lines, selection was applied in bulk and the relative proportion of different markers for each of the two parent lines was determined by pyrosequencing, using a method called linkage group selection [28]. Subsequent sequencing of the region that was enriched in resistant parasites identified two different mutations in a gene encoding a deubiquitinating enzyme, *pcubp-1*, suggesting that this gene could have resistance-conferring alleles [16,27]. Although there have not been many follow-up studies, the possible role of other genes in the ubiquitin pathway in artemisinin resistance means that *pcubp-1* remains a viable candidate. In fact, variant alleles of the *pfubp-1* gene in *P. falciparum* (E1528D) were significantly (*P* <0.001) more prevalent post-treatment in Kenyan children with reduced responsiveness to ACTs than in those who responded better to ACT [29].

## *In vitro* evolution and genome scanning

While the original linkage-group selection studies were underway, genome-analysis methods were becoming more tractable in terms of both cost and sensitivity. These methods allow researchers to find genes involved in resistance to various small molecules that have antimalarial activity by simply analyzing the complete genome of the multiple resistant clones created by *in vitro* evolution. Initially, tiling microarrays were designed to find both newly emerged SNVs and copy number variants (CNVs) [30,31]; later, these variants were identified by genome sequencing [32,33]. The approach was successful in part because multiple independent resistant lines were created, allowing identification of common genes that were mutated in all independent resistant lines. Although there was concern that it would be too difficult, it soon became evident that resistance to dihydroartemsinin (DHA) could be evolved in *P. falciparum* in the laboratory. Tucker and colleagues created several lines that were resistant to artelinic acid and artemisinin [34] and found potentially causative changes in candidate genes, including CNVs in *pfmdr1.* The results from full genome sequencing were published only in thesis format [35] and show that resistant lines acquired a handful of nonsynonymous mutations, including one in an uncharacterized protein on chromosome 13, *pf13_0238* (later renamed *PF3D7_1343700*), which would later come to be known as *kelch13* [36]. Other groups created parasites that were 25 times more resistant to DHA than the parental parasites [37]; these parasites showed further amplifications of a locus containing *pfmdr1*. Although gene expression microarray analysis was performed, the parasites were not subjected to whole-genome sequencing (WGS).

## Clinical resistance emerges

The search for markers that are associated with resistance became more urgent, and in some ways more feasible, when it became clear that parasites were developing resistance to artemisinin in the field. In 2008, a letter to the editor of the *New England Journal of Medicine* publicly documented the first clinical cases of suspected artemisinin resistance, in a patient population from Western Cambodia [38]. Noedl and colleagues [38] conducted clinical trials with artesunate monotherapy in 94 adults presenting with uncomplicated *P. falciparum* malaria in Battambang province. This study, looking at the presence of parasites in the blood after taking a standard dose, showed that artesunate alone had failed to clear parasites in two adults. The treatment of these two individuals was prolonged, but their infections were ultimately cleared. There is an active debate as to whether this situation should be best described as artemisinin tolerance, to distinguish it from that in which drug levels in the patient cannot be safely raised high enough to kill the parasites effectively and to prevent recrudescence [39-41]. For the sake of simplicity, the term ‘resistant’ will be used in this review.

In 2009, a more comprehensive study compared patient responses to artesunate monotherapy in Western Cambodia, Vietnam and north-western Thailand [17]. Measurements of parasite clearance times for 40 patients from each site demonstrated longer parasite clearance times in Cambodia than in Thailand. Furthermore, parasites taken from Cambodian patients into *in vitro* cultures demonstrated a significant IC_50_ increase for DHA, although not for chloroquine, mefloquine, or artesunate. The authors of this study noted that artemisinin administration in 2001 in Thailand was almost exclusively in the form of ACTs, while in Cambodia, 78% of artemisinin treatment had consisted of monotherapies, which can drive parasites to acquire resistance much more quickly. Although some sought to explain the longer parasite clearance time observed in Western Cambodia by an enrichment of possible human alleles (such as the hemoglobin E (HbE) polymorphism) in this region, studies showed that parasite genotype was more predictive than human genotype [42]. Some small, but statistically insignificant, differences in parasite-clearance times were nevertheless associated with some human alleles [42]. The human genotype theory became less likely as further studies were carried out. In 2012, resistance began to appear on the Thailand-Myanmar border where increases in parasite clearance time were quickly approaching those reported in western Cambodia [43]. At the same time, a report of artemisinin-resistant parasites in Myanmar was also published [44].

The existence of parasites with heritable resistance [42,45] spurred the design of parasite population genetic studies that could be used to map genes involved in resistance. In the absence of patient phenotype data, some groups sought simply to identify genomic regions under selection using large collections of existing parasites. It had been known for many years that there is linkage disequilibrium around genes involved with either chloroquine [46] or pyrimethamine resistance [47], and it was hypothesized that there might be genomic regions in disequilibrium that would correlate with artemisinin sensitivity.

In one study, 61 parasite lines were screened against the NIH Chemical Genomics Center Pharmaceutical Collection containing 2,816 compounds that are registered or approved for human or animal use. The parasite lines were genotyped and genotypes were examined for association with differential drug sensitivity to endoperoxides. Genes associated with responses to ART included *mal13p1.268* (a *Plasmodium* conserved protein), *pf11_0188* (a heat shock protein 90), *pfe0565w* (a conserved *Plasmodium* protein), *pf08_0130* (a ribosomal-RNA-processing WD-repeat protein), *pfa0655w* (SURFIN), and *pfi0355c* (an adenosine triphosphate-dependent heat shock protein) [48].

Mu and colleagues [49] subsequently genotyped 189 culture-adapted parasites collected from diverse locations, including 146 from Asia, using a custom-built Affymetrix molecular inversion probe 3 K malaria panel array with a coverage of approximately one single nucleotide polymorphism (SNP) per 7 kb. Their genome-wide scan for loci associated with responses to DHA, utilizing only Asian parasites, revealed novel loci on chromosome 1, 3 and 8 [49]. In another study with 45 cultured *P. falciparum* parasites from diverse geographical sources [50], some chromosomal regions (notably on chromosome 4) were found to be associated with increased sensitivity to DHA and artemisinin, but none of the associations were strong enough to be significant or worthy of follow-up. It should be noted that both of these studies provided strong evidence of selection around known resistance genes, such as *pfcrt*, *pfdfhr*, and *pfmdr1,* indicating that the overall method was working [49,50]. Although it is possible that artemisinin-resistance alleles might not have been suitably represented in the starting parasite populations, it is also possible that the standard IC_50_ assay that was used for phenotyping was not sufficiently sensitive. Artemisinin resistance is now considered easier to detect and quantify in cell culture using a ring-stage assay [51] of synchronized parasites (Figure 1).

These early studies clearly lacked both clinical phenotypic data and parasite samples with demonstrated resistance. To overcome this, studies were set up to recruit patients, to measure the amount of time needed to clear parasites after artemisinin monotherapy (compared with the standard ACT), and to obtain parasite material for genome analysis from areas such as Cambodia, where genetically determined resistance was present [43], as well as from control areas. The first major study, published in 2012, analyzed 91 parasite samples from Cambodia, Thailand, and Laos that were phenotyped for parasite clearance time [52]. The group utilized a custom Nimblegen genotyping array scoring both SNVs at a density of 1 per 500 bp as well as CNVs, with further fine-mapping using microsatellite analysis. The authors showed that although artemisinin resistance was probably not the result of a single originating event, either geographically or temporally, a clinical slow parasite clearance rate was strongly associated with a selective sweep on chromosome 13. Hypotheses about the actual gene involved were not resolved, although a 35 kb region on chromosome 13 (bases 1,759,466 to 1,794,766, PlasmoDB 11.1) was highlighted as a probable marker of resistance. Subsequent work by Ariey [36] would eventually show that the window was slightly too narrow, potentially because genotyping markers were too sparse in the region or alternatively because a genotyping marker was in a polymorphic sequence tract, which could distort the boundaries of a selective sweep.

Takala-Harrison and colleagues [53] genotyped parasites in 331 clinical infections from patients from Pailin, Cambodia, Wang Pha, Thailand and Bangladesh that had been phenotyped for parasite clearance time after artesunate monotherapy. An Affymetrix SNP array was used to analyze parasite genotypes at 8,079 positions. Modeling significant association with parasite clearance half-life, the time needed for parasitaemia to be reduced by half during the log-linear phase of parasite clearance [54], or parasite clearance time, four SNPs were identified on chromosomes 4, 10 and 13. Of these, two SNPs were calculated to be ‘located within a top-ranked signature of recent positive selection’. Both of these SNPs (MAL13-1718319 and MAL13-1719976) were found on chromosome 13, within 2,000 bp of each other; one was within *pf3d7_1343400* (formerly *mal13p1.216*, located between bases 1,714,443 to 1,719,255, PlasmoDB 11.1). This study was not designed to identify exact alleles causing resistance (as opposed to loci associated with resistance), but the authors further emphasized the importance of the 100-kb region on chromosome 13, although narrowly missing the probable gene with causative alleles.

Miotto and colleagues [55] sought to refine the mapping and identify possible causal SNPs in the locus under selection by genotyping 825 *P. falciparum* infections from 10 locations in West Africa and Southeast Asia. Infections were phenotyped for parasite clearance time after artesunate monotherapy in Southeast Asia and genotyped using short-read high-throughput sequencing on an Illumina platform. The authors showed that one resistant subpopulation of parasites from Southeast Asia (KH2) had essentially a single haplotype extending across half of chromosome 13, from 1.4 Mb to 3.4 Mb, which is strong evidence of a recent selective sweep. This group was able to postulate that the region was important, but even with genotyping at 86,158 coding SNPs, they were unable to perform further fine-scale population mapping without further sexual recombination between resistant and sensitive parasites to break up the interval.

## Combined approaches

It was not until very recently that a candidate artemisinin-resistance gene was identified with high confidence. In a report published in January of 2014 [36], Ariey and colleagues used a combination of next-generation WGS (approximately 500X) of an artemisinin-resistant line selected by continuous exposure to artemisinin for five years *in vitro* and population genetics studies. The genomic sequence from their laboratory-evolved artemisinin-resistant isolate was compared with that of an isogenic parent. After discarding emerged variants in multigene families, synonymous mutations, and alleles with mixed reads, Ariey and colleagues were able to identify eight non-synonymous candidate mutations in seven genes that had emerged during resistance selection. They retrospectively examined the times at which the mutations appeared in their line and concluded that a M476I coding change in *PF3D7_1343700*, a Kelch propeller domain-containing protein (K13), arose co-incident with the appearance of strong artemisinin resistance in their *in vitro* population and was most likely causative (Figure 1 and Box 3). Although it is too early to know the exact function of K13 in *P. falciparum*, studies in other organisms have sometimes shown a role in protein turnover (Box 3). The gene, *PF3D7_1343700* (bases 1,724,817 to 1,726,997, PlasmoDB 11.1) is notably very near the regions that had been identified as under selection by the population genetic studies [52,55]. To obtain further support, Ariey and colleagues sequenced the regions around the eight candidate SNPs in 49 culture-adapted isolates from Southeast Asia that had artemisinin sensitivity data associated with them, and showed that only mutations in *PF3D7_1343700* (including a C580Y change), were strongly associated with survival in the ring-stage assay (RSA) and with long parasite clearance half-life in patients, although they did not find the *in vitro*-derived M476I mutation in their samples. The authors analyzed the frequency of mutations in this gene in parasite samples from regions with and without resistance and found further association between resistance and this gene.

Another recent and comprehensive study by Ashley and colleagues [56] characterized parasite clearance half-life under the administration of artesunate monotherapy at ten sites, including seven spread throughout Southeast Asia. The authors also obtained the full sequence of *pfkelch13* using PCR amplification and Sanger sequencing (Figure 1). The authors, members of the Tracking Resistance to Artemisinin Consortium (TRAC), found long parasite clearance times in Indochina, but no significant resistance in a single patient from India, or anywhere in Africa. They found a strong association between mutations in *pfkelch13* and the artemisinin-resistance phenotype. Although this study involved assessments of parasite clearance in patients treated with artemisinin-based monotherapy, followed by standard combination therapy, treatment failure with artemisinin piperaquine ACTs have been reported in Cambodia [7,57]. One bright spot is that mutations in *pfkelch13* may also come with a cost to parasite fitness, and might be lost rapidly in populations in the absence of artemisinin selection.

## Genetic engineering

The propeller-domain mutations in *pf3kelch13* were only associated with resistance, although strongly, and were not shown to be causal. A genome-modification method, the CRISPR-Cas9 system, which was established for genome editing in other eukaryotes [58] and adapted to *P. falciparum* [59,60], was used to show definitively that one of the alleles and not some other second-site mutation caused resistance. The C580Y change was engineered into the Kelch propeller domain of *pf3kelch13* in the drug-sensitive NF54 background (of unknown origin). *In vitro* assays on two modified clones provided evidence of an increase in ring-stage resistance, providing additional support for the role of this gene [60]. Studies to assess the effect of introducing or removing the most prevalent *pfkelch13* mutations from clinical isolates are keenly awaited. These studies should firmly establish whether *pfkelch13* mutations do indeed confer resistance in clinical samples, although it is also possible that there are other, as yet unidentified, determinants.

## Future perspectives

One must keep in mind that the vast majority of malaria infections occur in Sub-Saharan Africa, where ACTs still appear to clear parasites quickly and where mutations in the *pfkelch13* have not yet appeared at elevated rates [56]. Longitudinal studies have not shown increased frequencies of mutations in *pfkelch13* in Ugandan children [61]. Although the Southeast-Asian alleles appear to be missing in Africa, African parasites have other *pfkelch13* alleles [62]. If clinical trials measuring parasite clearance time are used, there may be a problem with even quantifying the amount of resistance in Africa, where partial host immunity from repeated exposure may mask the loss of drug efficacy [63]. In fact, studies with Malian children show that antimalarial immunity correlates with fast artemisinin-induced parasite clearance [64]. In addition, artemisinins are typically given as a combination in Africa, and while there are reports of late-stage treatment failure after ACT use [7,57], there are not yet reports of early treatment failure and no deaths have yet resulted from ACT resistance. Chloroquine resistance is believed to have arisen just a few times in Africa and to have been imported from Asia in these cases [46]. Nevertheless, given that artemisinin resistance can be evolved in a tissue culture flask that contains many fewer parasites than a chronically infected human, the chance of independent *de novo* mutations emerging in Africa is high, especially as there has been a 30-fold increase in ACT usage worldwide (from 11 million courses to 331 courses) between 2006 and 2013 [2] as availability has increased. Furthermore, because mutations found from *in vitro* selection are not the same ones found in humans [37], there may be many ways to create resistance. Indeed, evidence suggests that artemisinin resistance has already emerged independently at least three different times in Southeast Asia alone [55].

Along with clinical monotherapy testing and cellular assays [51], the mutations in *pfkelch13* can now be used to study the spread of resistance and to identify zones where alternative therapies should be used [65]. However, many questions remain for scientists and clinicians. For example, are there other ways to create resistance besides mutations in *pfkelch13*? The laboratory-derived artemisinin-resistant lines created by Matthew Tucker had mutations in genes other than *pfkelch13*, including *pff0275c* (renamed *PF3D7_0605600*, a dinucleoside kinase) [35]. Are any of these additional mutations in some of the other chromosomal regions identified as under selection in population studies [52,53,55,56]? With which proteins does Pfkelch13p interact and would these also be resistant determinants? Will the mutations that have been identified in *pfkelch13* make parasites resistant against the synthetic endoperoxides, such as OZ439 [66]. Given that artemisinin has its greatest effect on trophozoite- and schizont-stage parasites [67], are there other genes, such as *pffalcipain-2* [68], that may be found mutated in field samples?

Studies of artemisinin resistance are already impacting patient treatment. In parts of Southeast Asia, older therapies are being reintroduced and patients are being admitted to hospitals so that their response can be monitored. There are also calls for focused and intensive plans to eliminate malaria from those regions were resistance has been observed to keep resistance from spreading [69]. While these measures may have an impact, it may be that the world health community will need to reduce reliance on this class of drugs. Luckily, many predicted that artemisinins would eventually lose their efficacy, and these predictions provided impetus to initiate searches for new classes of antimalarials, some of which are now in clinical trials.

## Box 1. Challenges to working with malaria parasites: a complex lifecycle and logistical barriers

Malaria parasites have a complex lifecycle (Figure 1). Although the parasite replicates asexually as a haploid organism in human and mosquito tissues, it has a sexual cycle with meiosis and a brief diploid phase, which occurs in the mosquito. The sexual re-assortment that occurs within mosquitoes is the basis for the genome-wide association studies of parasites in humans. Sexual crosses between resistant and sensitive parasites can be performed for *P. falciparum* and have been used to map drug-resistance genes in the past [70,71], but the method is not particularly accessible. Few researchers have access to all stages of the complex lifecycle, which is needed to complete genetic crosses. Although there are rodent models of malaria, which in some cases (such as *Plasmodium chabaudi*, *Plasmodium berghei*, and *Plasmodium yoelii*) can be more readily used in forward and reverse genetics, other human malaria parasites, such as *Plasmodium vivax*, cannot even be cultured long-term.

*P. falciparum* has an approximately 24 megabase haploid genome, notable for its extreme AT-richness [72]. Although malaria has been and continues to be a strong selective force on the human genome, the function of many of the predicted approximately 5,300 proteins encoded by the parasite genome can only be inferred from studies of orthologs in model organisms. A notable feature is that the genome bears long tracts of repetitive, recombinogenic sequence that may assist with immune evasion, but which makes genome manipulation and cloning challenging. Some of these recombinogenic tracts are within multigene families, some are intergenic, and some are within genes. For example, the amino terminus of PfKelch13 is predicted to have the low complexity protein coding sequence ‘NNNINHNNNNNNLTANNITNNLINNNMN’ within its first 200 amino acids (Figure 1). *In vitro* evolution studies have shown that repetitive sequences are more prone to mitotic gene conversion than sequences that do not contain repetitive sequences [73], but they are also more difficult to sequence and study. Outside of repetitive regions, the rate of mutation is probably similar to those found in other organisms [73].

Although the blood stages of *P. falciparum* can be maintained in cell culture using human erythrocytes obtained from donors, parasites cannot be as readily taken into cell culture for drug sensitivity testing. Furthermore, given that the disease can rapidly turn fatal, treatment recommendations may be made on the basis of the number of parasites that are PCR positive for a resistance marker in a region. The patient parasite clearance studies (Figure 1) in which parasite numbers are counted by simple light microscopy involve consented clinical trials in which patients agreed to be treated with a monotherapy (versus an ACT) initially but are closely monitored and then treated with a second drug or ACT. Although simple in design, these studies are relatively costly and are influenced by host factors, including a person’s immunity or whether the person has alleles that protect against malaria, such as the sickle cell allele, HbS. Individuals with this allele could theoretically clear parasites more quickly than those without. *In vitro* drug-sensitivity assays, in which parasites are incubated in the presence of increasing drug concentrations (to obtain an EC_50_), are more quantifiable (Figure 1) but may require more specialized laboratory equipment, such as incubators and tissue culture facilities. For artemisinin-resistance studies, a modified RSA in which parasites are first synchronized is typically used [51]. Genotyping parasites that have been phenotyped by both types of tests may be complicated by multiclone infections.

## Box 2. Artemisinins, their use and chemical background

There are a number of different artemisinin derivatives with antimalarial activities, including artesunate, dihydroartemisinin, and artemether (reviewed in [11]; Figure 2). Artemisinins are sesquiterpene lactones with a 1,2,4-trioxane core incorporating an endoperoxide linkage. Structure-activity relationship studies have shown that the active part of the molecule is the unusual endoperoxide bridge, and those synthetic molecules (for example, OZ439 and arterolane) that also bear this endoperoxide bridge are also very potent antimalarials [74]. There is substantial evidence that artemisinins form free radicals that attack various parasite proteins [75]. Parasites appear most susceptible to endoperoxides in the early ring stages of the parasite life cycle [68]. In fact, endoperoxides have reduced activity against stages of the life cycle during which hemoglobin digestion is not occurring, such as the hepatic stages [76], suggesting that hemoglobin digestion and the release of iron play a role in the action of the endoperoxide class. Indeed, inhibition of hemoglobinase activity with cysteine protease inhibitors, knockout of the cysteine protease *pffalcipain-2* by gene deletion, or direct deprivation of host cell lysate all significantly decrease the artemisinin sensitivity of *P. falciparum* [68].

Artemisinins are used in combination with partner compounds to prevent the development of resistance. Artesunate with amodiaquine, artesunate with mefloquine, artemether with lumefantrine, dihydroartemisinin with piperaquine, and artesunate with pyronaridine comprise what are generically known as ACTs. Although these are expensive relative to drugs composed of only older synthetic compounds such as chloroquine, they are nevertheless considered the gold standard for treating uncomplicated *P. falciparum* malaria because of their efficacy against multi-drug-resistant malaria and their rapid speed of action [77].

## Box 3. Kelch-domain proteins

Kelch-domain proteins are found throughout different phyla. Their name comes from the German word for chalice, and is derived from the appearance of the eggs of a mutant *Drosophila melanogaster* line first characterized by the developmental biologist and Nobel laureate Christiane Nusselin-Volhard [78]. Their disruption can give a wide variety of different phenotypes, although they are often found in complexes that perform ubiquitinylation in which the Kelch protein functions as the adaptor protein binding to the substrate that will be ubiquitinylated and marked for protein degradation. For example, in humans, the Kelch-like erythroid cell-derived protein with CNC homology [ECH]-associated protein 1, Keap1, which like Kelch13p contains a BTB dimerization domain, represses the antioxidant transcriptional response by facilitating the ubiquitinylation and proteosomal degradation of a key transcription factor, the NRF2 (nuclear factor erythroid 2-related factor 2 (Nrf2)) transcription factor [79], in the absence of oxidative stress [80]. In human hepatocytes, modulation of Keap1 activity also alters the cell cycle, delaying S phase entry [81].

Notably, artemisinin treatment causes ring-stage parasites, which are substantially less susceptible to the killing action of the drug [67], to enter a dormant phase [34,82], and at least some resistant parasites seem able to recover from dormancy after treatment and begin growing again [82]. Some genetically resistant parasites from Southeast Asia, where *pfkelch13* mutations are common, have an extended ring stage and longer cell cycle [83].

**Figure 3:**
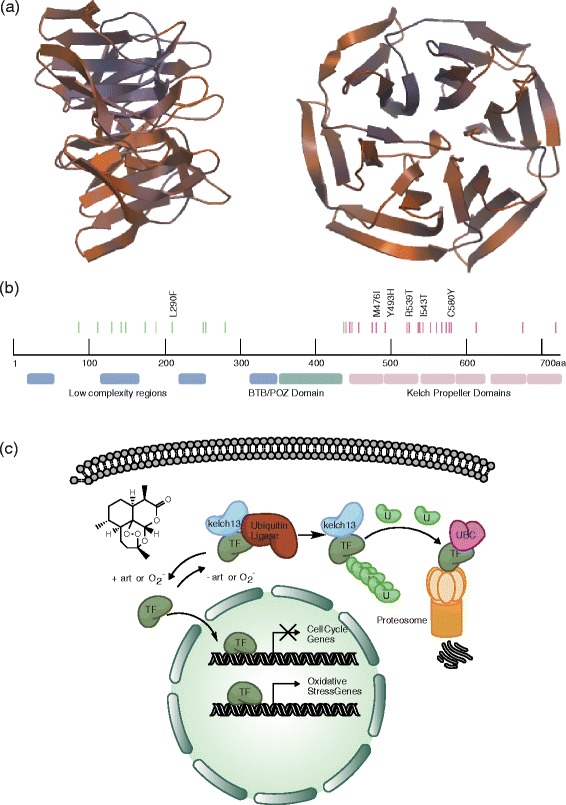
**Kelch structure, the position of mutations in the** ***pfKelch13*** **gene, and a theoretical functional model. (a)** Homology model of PfKelch13p (amino acids 444 through 723) generated using SWISS-MODEL and the human Kelch-like protein 12 crystral structure (2vpj.1.A) as a template. Two rotation views are shown. **(b)** Predicted domains and locations of mutations in *pfkelch13* (*PF3D7_1343700*) identified either *in vitro* [35,36] or *in vivo* [36,56]. Mutations in pink are in the Kelch domain modeled above while green ones are in the predicted regions with more ambiguous function. Mutations mentioned in the text are specifically indicated. In addition to Kelch domains, PfKelch13p contains a BTB domain, typically involved in dimerization. **(c)** Theoretical model of PfKelch function in artemisinin resistance. See Box 3 for details. TF, transcription factor.

Figure 3 shows a hypothetical model based on existing data. In the presence of artemisinin, free radicals are present that cause a subset of ring-stage parasites to enter a transcriptionally or epigenetically regulated dormant phase modulated by possible nuclear proteins (transcription factors (TFs)) that respond to oxidative stress. In most human infections, the red cells containing these dormant parasites are eventually cleared with the assistance of the immune system and the spleen (as human malaria is not always fatal in the absence of chemotherapy), resulting in a cure. Mutations in *pfkelch13* might prime the parasite to respond to oxidative stress while simultaneously increasing the time spent in the less susceptible times of the cell cycle. These responses would result in a higher proportion of dormant parasites that have the potential to survive and recrudesce. In the absence of *pfkelch* mutations, the TF could be ubiquitinylated in the cytoplasm and degraded, a process potentially mediated by PfKelch13p. A ubiquitin carboxy (UBC) terminal hydrolase could be involved in recycling ubiquitin - its disruption might also drive the putative TF to a de-ubiquitinylated state. A role in ubiquitin-dependent protein turnover or regulation could explain why rodent malaria parasites that are exposed to artesunate acquire mutations in a gene encoding the deubiquitinating enzyme, *pcubp1* (*pf3d7_0104300* a ubiquitin carboxyl-terminal hydrolase 1 in *P. falciparum*) [27]). Although this seems the most plausible model, others are possible: for example, *pfkelch13* also encodes a weak CAF1 150 domain, named after a domain found in chromatin assembly factor subunit 150 [80], the complex that loads histones onto newly replicated DNA. Thus *pfkelch13* could also directly impact gene silencing and transcriptional regulation.

## Abbreviations

ACT: artemisinin-based combination therapies
CNV: copy number variant
DHA: dihydroartemisinin
IC50: inhibition constant 50
PfCRT: *P. falciparum* chloroquine resistance transporter
PfMDR1: *P. falciparum* multidrug resistant protein 1
RSA: ring-stage assay
SNP: single nucleotide polymorphism
SNV: single nucleotide variant
SNV: single nucleotide variant
TRAC: Tracking Resistance to Artemisinin Consortium
WGS: whole-genome sequencing
